# Anticoronavirus Evaluation of Antimicrobial Diterpenoids: Application of New Ferruginol Analogues

**DOI:** 10.3390/v15061342

**Published:** 2023-06-09

**Authors:** Mihayl Varbanov, Stéphanie Philippot, Miguel A. González-Cardenete

**Affiliations:** 1 Université de Lorraine, CNRS, L2CM, F-54000 Nancy, France; 2 Laboratoire de Virologie, CHRU de Nancy Brabois, 54500 Vandoeuvre-lès-Nancy, France; 3 Instituto de Tecnologia Química (UPV-CSIC), Universitat Politècnica de Valencia-Consejo Superior de Investigaciones Científicas, Avenida de los Naranjos s/n, 46022 Valencia, Spain

**Keywords:** antimicrobial, coronavirus, abietane, dehydroabietylamine, phenol, phthalimide

## Abstract

The abietane diterpene (+)-ferruginol (**1**), like other natural and semisynthetic abietanes, is distinguished for its interesting pharmacological properties such as antimicrobial activity, including antiviral. In this study, selected C18-functionalized semisynthetic abietanes prepared from the commercially available (+)-dehydroabietylamine or methyl dehydroabietate were tested in vitro against human coronavirus 229E (HCoV-229E). As a result, a new ferruginol analogue caused a relevant reduction in virus titer as well as the inhibition of a cytopathic effect. A toxicity prediction based on in silico analysis was also performed as well as an estimation of bioavailability. This work demonstrates the antimicrobial and specifically antiviral activity of two tested compounds, making these molecules interesting for the development of new antivirals.

## 1. Introduction

Viral diseases are a significant global health problem with millions of infections annually and insufficient antiviral drugs available. For viruses such as Zika (ZIKV), Ebola (EBOV), and many others, there is no available explicit antiviral treatment currently [1]. These health problems have led to the (re)evaluating of traditional medical approaches in order to focus on new molecules or new mechanisms of action. Therefore, naturally based medicines may be appropriate alternatives for treating viral infections [2]. In fact, about fifty percent of clinical anti-infective medications, of which one third are antiviral drugs, include either natural products (NPs), molecules derived from NPs or inspired by NP structures [3].

Viral respiratory diseases cause life-threatening infections in millions of patients worldwide every year. Human coronaviruses (HCoVs) are accountable for steady epidemic outbreaks, thus representing a global public health concern. HCoVs are enveloped, positive single-stranded RNA viruses which provoke a large portion of upper and middle respiratory tract infections, such as cold, bronchitis, and pneumonia. Coronavirus infection can be especially severe and fatal for newborns, young infants, the elderly, or immunosuppressed people [4].

The COVID-19 pandemic caused by SARS-CoV-2 has infected more than 700 million people and caused nearly seven million deaths [5]. At present, few antiviral drugs against SARS-CoV-2 are in or close to clinical use. These include molnupiravir (Merck), and Paxlovid (Pfizer). Paxlovid is a combination of the main viral protease Mpro (also known as 3-chymotrypsin-like protease, 3C-like protease or 3CLpro) inhibitor nirmatrelvir (PF-07321332) and ritonavir (a CYP3A4 inhibitor that slows down the clearance of nirmatrelvir) [6]. The potential of drug–drug interactions from ritonavir, however, may limit its use by many patients [7]. Additionally, it seems that the efficacy of the treatment with molnupiravir is low since it requires a high intake of pills (40 in 5 days). Given the spread and severity of the disease, it is crucial to develop efficient treatments and rapidly available solutions that can supplement active immunization efforts, which are still challenged by high viral transmissibility, re-infection, and immune escape variants.

In 2021, Skaltsounis et al. [8] reviewed natural and nature-derived products targeting human coronaviruses since their discovery in the 1960s, and realized the scarce research on this subject. However, it is known that a number of diterpenes are potential antiviral agents, including towards coronaviruses [9]. Very recently, Jantan et al. [10] have reviewed the antiviral effects of a number of phytochemicals against SARS-CoV-2, including virtual screening carried out over the last three years.

**Figure 1:**
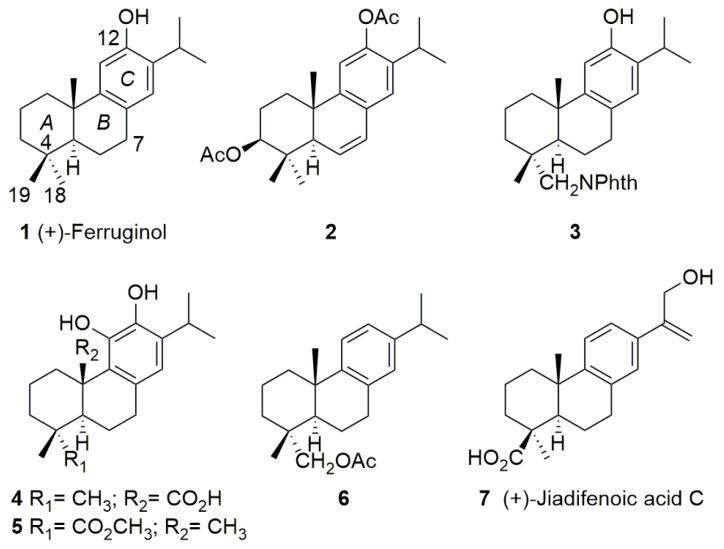
Natural and semisynthetic antiviral abietanes **1**–**7**.

The abietane-type diterpenoids are naturally occurring compounds isolated from plants of different families such as *Araucariaceae*, *Cupressaceae*, *Phyllocladaceae*, *Pinaceae*, *Podocarpaceae*, *Asteraceae*, *Celastraceae*, *Hydrocharitaceae*, and *Lamiaceae* [11]. These metabolites show an extensive range of promising pharmacological activities, including antimicrobial properties [11]. Several semisynthetic derivatives have also displayed interesting pharmacological properties [12]. For example, natural (+)-ferruginol (**1**) and diacetate (**2**) (3β,12-diacetoxyabieta-6,8,11,13-tetraene) have shown important anti-SARS-CoV activities with a high cytopathogenic effect (CPE) reduction and effective replication inhibition (SARS-CoV, EC_50_ = 1.39 and 1.57 μM, respectively, in Vero E6 cells) [13]. Wen et al. (2007) [13] found that ferruginol (**1**) did not show inhibition for SARS-CoV 3CL protease (3CLpro), a key target for antiviral drug design, below 100 μM; later, other researchers reported significantly better inhibitory effects on 3CLpro (IC_50_ = 49.6 μM) [14]. Additionally, the semisynthetic phthalimide ferruginol analogue **3** (Figure 1) has shown anti-dengue and anti-herpes properties [15], and anti-Zika activity against Brazilian strains [16] as well as anti-Zika and anti-chikungunya activities against Colombian strains [17]. Carnosic acid (**4**) has displayed inhibitory effects on human immunodeficiency virus 1 (HIV-1) protease and HIV-1 virus [18], and on human respiratory syncytial virus replication [19]. Its semisynthetic analogue **5**, however, demonstrated a better anti-herpes profile than carnosic acid itself [20]. Semisynthetic dehydroabietinol acetate (**6**) showed mild anti-herpes activity [21]. In recent years, several C19-functionalized abietane-type acids, i.e., jiadifenoic acid C (**7**), and its derivatives isolated from *Illiciaceae* plants (jiadifenoic acids from the roots of *Illicium jiadifengpi*) have exhibited important anti-Coxsackie virus B activities [22].

**Scheme 1:**
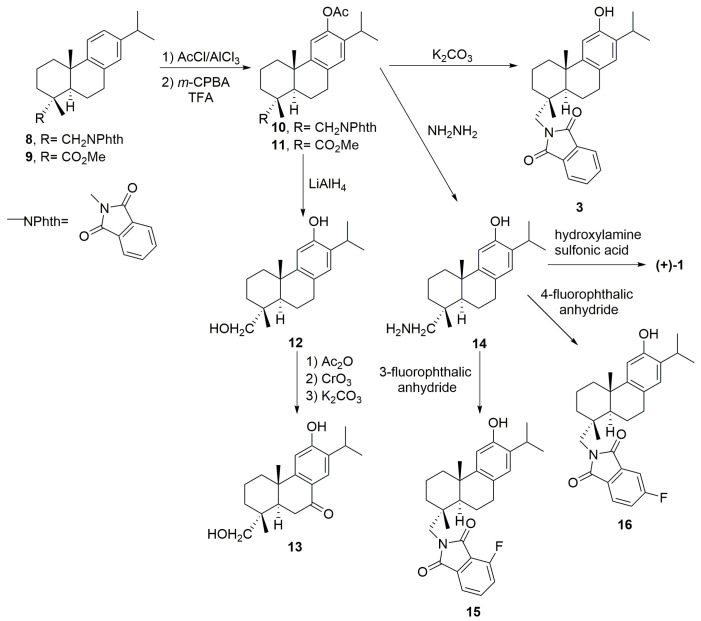
Synthetic sequence for the preparation of tested molecules **1**, **3**, and **12**–**16**.

Based on this background of antimicrobial activities of abietane-type diterpenoids, in this work, we have extended the knowledge of antiviral activities of several C18-functionalized ferruginol analogues. The current research aimed to find potential molecules to inhibit the old coronavirus associated infection as a model for broad-spectrum anticoronavirus agents. Thus, we report on the in vitro antiviral study of ferruginol (**1**), ferruginol analogues **3** and **12**–**16** (Scheme 1) against *human coronavirus 229E*.

## 2. Materials and Methods

### 2.1. Chemistry: General Experimental Procedures

Specific rotation was measured using a 10 cm cell in a Jasco P-2000 polarimeter in DCM. NMR data were collected on a 300 MHz spectrometer. All spectra were recorded in CDCl_3_ as solvent. Reactions were monitored by TLC using Merck silica gel 60 F254 (0.25 mm-thick) plates. Compounds on TLC plates were visualized under UV light at 254 nm and by immersion in a 10% sulfuric acid solution and heating with a heat gun. Purifications were performed by flash chromatography on Merck silica gel (230–400 mesh). Commercial reagent grade solvents and chemicals were used as purchased. Combined organic extracts were washed with brine, dried over anhydrous MgSO4, filtered, and concentrated under reduced pressure.

(+)-Dehydroabietylamine (ca. 60%) was purchased from Aldrich (Saint Louis, MO, USA) and (−)-abietic acid >70% from TCI Europe (Zwijndrecht, Belgium). Carbon numbering of compounds matched to that of natural products. All known compounds prepared in this work displayed spectroscopic data in agreement with the reported data [17,23,24,25]. Purity of final compound was 95% or higher. Copies of NMR spectra for new compounds **15** and **16** are available in the supplementary information.

### 2.2. Synthesis

*18-Aminoferruginol or 12-hydroxydehydroabietylamine* (**14**).

To a stirred yellow suspension of phthalimide **10** (3.5 g, 7.4 mmol) [17] in ethanol (65 mL) excess hydrazine monohydrate (50%, 2.8 mL, 44.4 mmol) was added, turning it to pink. Then, it was refluxed for 5 h. Then, without cooling, a white solid was filtered off and washed with fresh ethanol (3 × 20 mL). The filtrate was concentrated to give crude amino-phenol **14** (6.7 g). This crude solid was treated with 80 mL of aqueous 2 M NaOH and stirred for 2 h 30 min at 700 rpm. The resulting basic solution was neutralized with approx. 57.5 mL 10% HCl, and then extracted with dichloromethane (5 × 60 mL). The extract was dried over MgSO_4_ and concentrated to give 2.10 g (94%) of amino-phenol **14** as a pale solid which was used without further purification with spectral data in agreement with the reported data [23].

*12-Hydroxy-N,N-(3-fluorophthaloyl)dehydroabietylamine* (**15**).

A mixture of 3-fluorophthalic anhydride (120 mg, 0.73 mmol) and 12-hydroxydehydroabietylamine **14** (200 mg, 0.66 mmol) in AcOH (2.5 mL) was refluxed for 2.5 h, then cooled to rt and 30 mL of water was added. The resulting solid was filtered off, washed with water, and dried under vacuum to give compound **15** (150 mg, 50%) as a beige solid: [α]^20^_D_–20.5 (c 1.0, CH_2_Cl_2_); ^1^H NMR (300 MHz) δ 7.73–7.62 (2H, m), 7.35 (1H, t, *J* = 9.0), 6.86 (1H, s), 6.60 (1H, s), 3.67 (1H, d, *J* = 13.8), 3.49 (1H, d, *J* = 13.8), 3.10 (1H, sept., *J* = 6.9), 2.95–2.89 (2H, m), 2.28–2.10 (2H, m), 1.85–1.62 (3H, m), 1.52–1.30 (4H, m), 1.22 (3H, d, *J* = 6.9), 1.22 (3H, s), 1.20 (3H, d, *J* = 6.9), 1.04 (3H, s); ^13^C NMR (75 MHz) δ_C_ 168.1 (d, *J*_C,F_ = 3), 165.9 (s), 157.4 (d, *J*_C,F_ = 264), 150.6 (s), 148.2 (s), 136.4 (d, *J*_C,F_ = 7.5), 134.2 (d, *J*_C,F_ = 1.5), 131.6 (s), 127.2 (s), 126.8 (d), 122.3 (d, *J*_C,F_ = 20), 119.4 (d, *J*_C,F_ = 4), 117.7 (d, *J*_C,F_ = 12), 110.5 (d), 49.0 (t), 45.1 (d), 39.4 (s), 38.1 (t), 37.5 (s), 36.9 (t), 29.3 (t), 26.8 (d), 25.7 (q), 22.7 (q), 22.6 (q), 19.5 (t), 19.1 (q), 18.5 (t); ^19^F NMR (282 MHz) δ_F_ − 113.0; HRMS (ESI) *m*/*z* 450.2441 [M+1]+, calcd for C_28_H_33_FNO_3_: 450.2444; Anal. calcd. for C_28_H_32_FNO_3_: C, 74.8; H, 7.2; N, 3.1; Found: C, 74.5; H, 7.3; N, 3.1.

*12-Hydroxy-N,N-(4-fluorophthaloyl)dehydroabietylamine* (**16**).

A mixture of 4-fluorophthalic anhydride (332 mg, 1.99 mmol) and 12-hydroxydehydroabietylamine **14** (305 mg, 1.01 mmol) in pyridine (2.5 mL) was heated at reflux for 4 h, then cooled to rt and 15 mL of water was added, followed by extraction with diethyl ether (5 × 6 mL). The combined extract was washed with 10% HCl (2 × 5 mL), water (2 × 5 mL) and brine (5 mL), dried over MgSO_4_, and concentrated under vacuum. The resulting beige foamy residue (360 mg) was purified by column chromatography eluting with *n*-hexane-ethyl acetate 7:3 to give compound **16** (256 mg, 56%) as a pale oil which became a light beige solid after trituration with *n*-hexane: [α]^20^_D_–29.6 (c 1.0, CH_2_Cl_2_); ^1^H NMR (300 MHz) δ 7.81 (1H, dd, *J* = 8.1, 4.5), 7.48 (1H, dd, *J* = 7.2, 2.1), 7.35 (1H, ddd, *J* = 8.4, 8.4, 2.2), 6.86 (1H, s), 6.59 (1H, s), 4.58 (1H, s, OH), 3.67 (1H, d, *J* = 14.0), 3.50 (1H, d, *J* = 14.0), 3.10 (1H, sept., *J* = 6.9), 2.92–2.89 (2H, m), 2.24–2.06 (2H, m), 1.87–1.59 (4H, m), 1.52–1.30 (4H, m), 1.23 (3H, d, *J* = 6.9), 1.21 (3H, d, *J* = 6.9), 1.21 (3H, s), 1.04 (3H, s); ^13^C NMR (75 MHz) δ_C_ 168.2 (s), 167.9 (d, *J*_C,F_ = 3), 166.3 (d, *J*_C,F_ = 255), 150.6 (s), 148.2 (s), 134.8 (d, *J*_C,F_ = 9), 131.6 (s), 127.7 (d, *J*_C,F_ = 3), 127.2 (s), 126.8 (d), 125.6 (d, *J*_C,F_ = 10), 120.9 (d, *J*_C,F_ = 24), 111.0 (d, *J*_C,F_ = 25), 110.5 (d), 49.1 (t), 45.1 (d), 39.4 (s), 38.1 (t), 37.5 (s), 36.9 (t), 29.3 (t), 26.8 (d), 25.7 (q), 22.7 (q), 22.6 (q), 19.5 (t), 19.1 (q), 18.5 (t); ^19^F NMR (282 MHz) *δ*_F_ − 102.0; HRMS (ESI) *m*/*z* 450.2431 [M+1]^+^, calcd for C_28_H_33_FNO_3_: 450.2444; Anal. calcd. for C_28_H_32_FNO_3_: C, 74.8; H, 7.2; N, 3.1; Found: C, 74.4; H, 6.8; N, 2.9.

### 2.3. Anti-HCoV 229E Assay

#### 2.3.1. Cell Culture and Virus

For virus production and for the antiviral assay MRC-5 human lung fibroblast were used (ECACC, ref 05090501, Sigma-Merck, Saint Quentin, France). The cells were grown in antibiotic-free Minimum Essential Medium (MEM, M4655, Sigma-Merck, Saint Quentin, France) supplemented with 10% fetal calf serum (FCS, CVFSVF00-01, Eurobio, Les Ulis, France), 2 mM glutamine (G7513, Sigma-Merck, France), 1 mM sodium pyruvate (SH30239.01, Hyclone GE Healthcare, Logan, UT, USA), and 1 × MEM non-essential amino acids (11140-035, Gibco Life Technologies) (i.e., growth medium) at 37 °C in 5% CO_2_. For the antiviral assays the medium was supplemented only with 2% FCS (i.e., maintenance medium). The cultures were maintained at 33 °C in humidified 5% CO_2_ atmosphere.

The human coronavirus HCoV 229E (PHE/NCPV 0310051v) was produced and quantified in MRC-5 cells. Initial virus titers were calculated as 50% cell-culture infectious doses (CCID_50_/mL), defined as the dilution of the virus required to infect 50% of the cell culture, according to Reed and Muench (1938) [26]. All virus stocks were stored at −70 °C until further use. The infectivity of the viral samples was titrated on 96-well microtiter plates containing about 80% confluent MRC-5 cells. A volume of 100 μL of serial 10-fold dilutions of the virus from 10^−1^ to 10^−3^ in DMEM medium with 2% FCS was added to the MRC-5 cells, in 8 replicas. The infected cells were incubated at 33 °C in 5% CO_2_ for 6 days. The appearance of cytopathogenic effect (CPE) was recorded daily.

The lyophilized compounds were dissolved at 50 mg/mL in DMSO (D8418, Sigma-Merck, France) at room temperature. Stock solutions of compounds were kept at 4 °C until used. The concentration of DMSO in biological assays was of 0.1%, including in cell controls.

#### 2.3.2. Cytotoxicity

For cytotoxicity evaluation, MRC-5 cells were seeded at 15 × 10^3^ cells per well in 96-well tissue-culture plates. The DMSO-dissolved compounds were diluted in MEM complete medium with 2% FCS. At 24 h after the seeding, a limited volume of 100 µL of each compound were added to the cell monolayers at increasing concentrations (from 3.6–82.7 µM). The plates were then incubated for 72 h at 37 °C in a 5% CO_2_ atmosphere. For assessment of the cell viability the MTT assay was used [27]. Percentages of survival compared to control were calculated and the maximal concentration with no cytotoxic effect was determined. The cytotoxicity assays were performed in triplicates. Values are presented as means of these independent experiments.

#### 2.3.3. Antiviral Assay

The antiviral activity of the compounds was evaluated using the reduction in the viral titer and the reduction in the virus-induced cytopathogenic effect (CPE), characterized mostly by extensive host cell death and degradation of the cell monolayer.

The cells were seeded in 96-well plates at 15,000 cells/well. After 24 h of incubation, two different multiplicities of infection (MOI) of the virus (viral batch of 22 February 2021, 2.4 × 10^4^ IP/mL) were evaluated, in cascade, with serial dilutions at 1/10 in the cell culture medium (2% fetal calf serum) containing or not the compound to be tested (at 3 μg/mL final concentration). Cell plates were emptied, then the cell monolayers were treated with 100 μL of the dilutions (one column per condition, n = 8). An untreated control and a 0.1% DMSO control were added to each plate. The plates were incubated at 33 °C for 72 h. The cytopathic effect of the virus was evaluated by reading the plate under a microscope (Zeiss, Rueil-Malmaison, France) and the viral titers were determined according to the Reed and Muench method [26]. The plates were then stained with crystal violet and the cytopathogenic effect of each well was calculated using a plate reader, measuring the optical density of each well at 540 nm.

Wells were tested the same way for each assay, including control or blank (n = 8). The test wells were assessed for the determination of viral CPE post-infection when cell destruction of infected untreated cultures was at its maximum. The median tissue culture infectious dose (TCID_50_) was determined, and titrations were performed in triplicate. Titres were calculated as (TCID_50_)/mL. The antiviral assays were performed in triplicates. Classic tissue culture infectious dose 50% (TCID_50_/mL) were performed in triplicates of a single experiment.

### 2.4. In Silico Simulations

#### 2.4.1. Calculation of Molecular Properties (Drug-Likeness)

The structures of tested compounds were manually drawn in ChemDraw Professional 15.0 software (PerkinElmer Informatics, Inc., Waltham, MA, USA), and the SMILES notation was obtained for each molecule. To calculate the molecular polar surface area [28] and the parameters of Lipinski’s rule of five (see Table 1) [29,30], the SMILES notation in Molinspiration online 2023 software (available from https://www.molinspiration.com/cgi-bin/properties, accessed on 27 January 2023, Molinspiration Cheminformatics, Slovensky Grob, Slovak Republic, 2023, https://www.molinspiration.com) was employed.

#### 2.4.2. Toxicity Prediction (GUSAR)

Compounds **1**, **3**, and **12**–**16** were investigated for acute rat toxicity properties in silico (see Table 2). The acute toxicity in rodent models of the tested compounds were predicted by GUSAR [31]. The analysis was based on the quantitative neighborhoods of atom descriptors and the prediction of activity spectra for substances algorithm. It correlates the results with the SYMYX MDL toxicity database and further classifies the tested compounds according to the Organization for Economic Co-operation and Development (OECD) manual.

Toxic doses are expressed as median lethal dose, representing the dose of the compound that is lethal to 50% of the experimental animals exposed to it. The LD_50_ values are expressed as the weight of the chemical per unit of body weight (mg/kg). The compounds were classified based on the toxicity scale used in the Gosselin, Smith and Hodge scale: class 1 (LD_50_ ≤ 5 mg/kg) being super toxic, class 2 (5 < LD_50_ ≤ 50 mg/kg) being extremely toxic, class 3 (50 < LD_50_ ≤ 500 mg/kg) being very toxic, class 4 (500 < LD_50_ ≤ 5000 mg/kg) being moderately toxic, class 5 (5000 < LD_50_ ≤ 15,000 mg/kg) being slightly toxic, and practically non-toxic compounds with LD_50_ > 15,000 mg/kg [32].

## 3. Results

The tested compounds **1**, **3** and **12**–**14** are known and were obtained following synthetic routes previously reported, as follows (see above Scheme 1): compounds **1** and **3** following our recently optimized conditions [17] through intermediate **10**; meanwhile compounds **12** and **13** where obtained from intermediate **11** [24]; for the synthesis of compound **14** (12-hydroxydehydroabietylamine) we optimized our previous work [23] starting from **10**, changing slightly the protocol to achieve a better yield. Compound **14** was the intermediate for novel compounds **15** and **16**. In particular, compounds **1**, **3** and **14**–**16** were obtained from commercially available (+)-dehydroabietylamine, which by condensation with phthalic anhydride gives phthalimide **8** [17,25]. To make the C-12 phenolic moiety Friedel–Crafts and Baeyer–Villiger reactions were used (Scheme 1). Thus, Friedel–Crafts acylation of **8** followed by Baeyer–Villiger oxidation gave acetate **10** [25], which by mild basic methanolysis afforded ferruginol analogue **3** named 18-(phthalimid-2-yl) ferruginol [17]. By contrast, the treatment with hydrazine of compound **10** furnished phenol-amine **14** which served as an intermediate for the synthesis of (+)-ferruginol (**1**), by deamination [23], and fluorinated phthalimides **15** and **16** by condensation with either 3-fluorophthalic anhydride or 4-fluorophthalic anhydride, respectively. Similarly, starting from methyl dehydroabietate (**9**) [21], prepared from (−)-abietic acid by esterification and aromatization with Pd/C catalyst, followed by Friedel–Crafts reaction and Baeyer–Villiger oxidation, gave acetate **11** which was first converted into alcohol **12** by reduction with LiAlH_4_, and then into keto-alcohol **13** in a three-step sequence [24].

**Figure 2:**
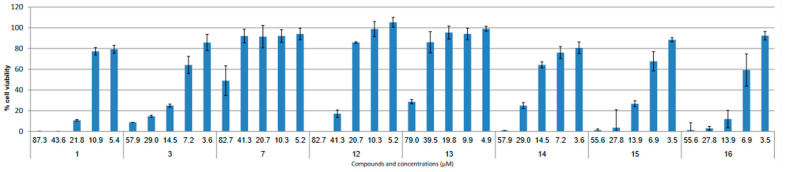
Cell viability after 72 h incubation with the compounds at 3.6–82.7 µM (n = 3).

**Figure 3:**
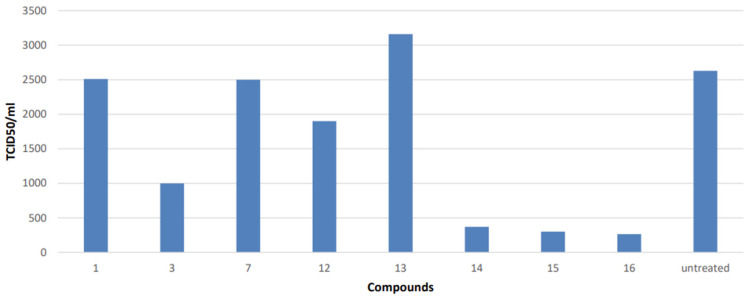
Effect of the compounds on the viral titer of HCoV 229E (n = 1).

**Figure 4:**
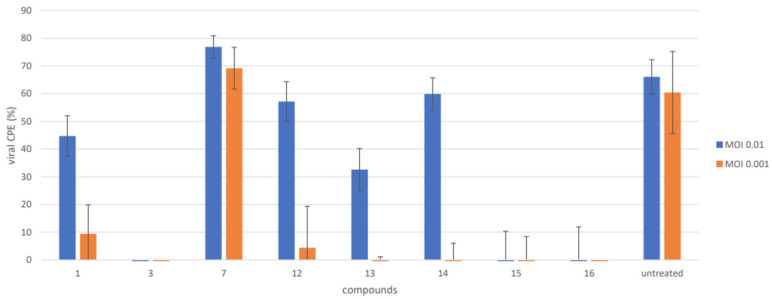
Antiviral effect based on the variation of the cell monolayer density and on the HCoV 229E-induced CPE (n = 3).

The antiviral activities of the compounds were evaluated against a respiratory virus, human coronavirus 229E (*HCoV 229E*). These compounds were investigated at different concentrations depending on cytotoxicity (Figure 2). The antiviral of the compounds against *HCoV 229E* were determined according to the Reed and Muench end-point dilution method [26] and are shown in Figure 3 and Figure 4 (see below). Three of the compounds reduced the viral titer, with low to no cytotoxicity at 3 µg/mL (Figure 2). The compound **12** moderately reduced the viral titer with close to 0.5-log, while the compounds **14**, **15** and **16** showed significant reduction in the viral titer with approximately 2-logs (Figure 3). The analysis of the reduction in the viral-induced cytopathogenic effect (CPE) confirmed the antiviral activity of compound **16** (at 3.0 µg/mL or 6.7 μM), where the cytopathogenic effect was totally abolished at all the applied MOIs (0.01–0.001). Compounds **3** and **15** also showed important reductions in the viral titer, similar to the one induced by **16**, but they also induced modification of the cell morphology in photonic microscope observation. The rest of the compounds also produced important reductions in the viral CPE, but mostly at lower MOIs (0.001), except jiadifenoic acid C (**7**) which showed no activity (Figure 4).

In silico computation is, nowadays, an important part of the drug design development process. Using the Molinspiration Cheminformatics software (Molinspiration, accessed on 27 January 2023, Slovensky Grob, Slovak Republic, 2023, http://www.molinspiration.com), through an easily obtained SMILES notation for each molecule, we can calculate certain very useful molecular properties such as the Lipinski’s rule of five [29]. The compounds **1**, **3**, **12**, **13**, **14**, **15** and **16** were subjected to this software and we obtained very promising results of drug-likeness (Table 1).

The computational prediction of compound toxicities in drug design allows the quick estimation of the lethal doses in animals, and avoids the use of animal experiments. The rat acute toxicity of the compounds **1**, **3**, **12**, **13**, **14**, **15** and **16** was predicted by the in silico software tool GUSAR (General Unrestricted Structure—Activity Relationships, version 16.0) [31] on the basis of PASS (Prediction of Activity Spectra for Substances) technology [33,34]. The quantitative in silico prediction was applied to four different types of administration: oral, intravenous (IV), intraperitoneal (IP), and subcutaneous (SC). As is shown in Table 2, the differences in the predicted LD_50_ values obtained for the four different routes, based on the structural formulae of the tested compounds, are reported along with the acute rodent toxicity classification of the compounds. Extreme/super toxicities (class 1) are documented for compound **1** for all routes of administration, and for compound **3** in the case of intravenous administration, where the value of 0.00 indicates that the compound falls out of the domain of applicability of the proposed route of administration. Compounds **13**, **14**, **15** and **16** are to be considered as very toxic (class 3) in the event of intravenous administration. Depending to the route of administration (mainly IP, oral and SC) compounds **3**–**16** were estimated to have moderate (class 4), slight (class 5), and practically non–toxic behavior based on the calculated LD_50_ values.

## 4. Discussion

Nature provides a huge diversity of chemicals to explore and develop drugs for the treatment of various ailments including viral diseases; however, it is difficult to estimate adverse effects since often the available studies are in vitro models [35]. Natural products and their derivatives against coronavirus have been reviewed recently, showing promising results [8,36]. To date, most of the studies have focused on SARS-CoV models with proposed mechanisms of action associated with 3CL protease (Mpro) [37]. We reported, in 2016, that several abietane-type diterpenoids exhibited antiviral activity in viral models of dengue with EC_50_ < 5.0 μM and herpes with EC_50_ < 20 μM [15]. The phthalimide-ferruginol analogue, compound **3**, was the most promising and potent. In recent years, we have explored the antiviral properties of other semisynthetic abietane diterpenoids, including compound **3**, in models of Brazilian Zika virus [16] and Colombian Zika and Chikungunya viruses [17]. Ferruginol (**1**), compound **3** as well as some of the ferruginol analogues herein studied are characterized by a 2-isopropylphenol moiety which is typical of the monoterpenoid thymol (2-isopropyl-5-methylphenol). Thymol and its source essential oil have been also investigated as antiviral agents [38]. The precedent that ferruginol (**1**) had demonstrated a high cytopathogenic effect (CPE) reduction and effective replication inhibition for SARS-CoV (EC_50_ = 1.39 μM) [13] encouraged us to study this molecule as well as other readily synthesized analogues in other coronavirus models. Recently, some in silico studies of abietanes (for example, sugiol or 7-oxoferruginol) have demonstrated the potential inhibition of 3CLpro (Main protease, Mpro) of SARS-CoV-2 [39] and, more importantly, some extracts of medicinal plants containing abietanes (Rosemary, i.e., (+)-carnosic acid) have demonstrated anti-SARS-CoV-2 activity in cell assays [40]. Tanshinones are related abietane diterpenoids first isolated from the roots of *Salvia miltiorrhiza* “tanshen”, a well-known Traditional Chinese Medicine [41]. Park et al. [41] investigated, in 2012, several isolated tanshinones on SARS-CoV 3CLpro with very promising results. Recently, tanshinone IIA has shown anti-SARS-CoV-2 activity with IC_50_ of 4.08 ng/μL [42]. The studies in this field are rising as other semisynthetic abietane derivatives have also shown, very recently, anti-SARS-CoV-2 pseudovirus activity [43]. Our results with 12-hydroxydehydroabietylamine (**14**) and 12-hydroxy-N,N-(4-fluorophthaloyl)dehydroabietylamine (**16**) point out that these molecules do reduce the cytopathogenic effect of human CoV 229E and possess molecular characteristics useful for further studies of drug development such as drug-likeness and safety. On the one hand, compound **16** is a fluorinated analogue which at 6.7 μM removed all CPE with a reduction in the viral titer of 2-logs approximately. Its molecular structure draws only one violation of the well-known Lipinski’s rule of five, in particular, its calculated partition coefficient which may lead to the design of pro-drugs for enhanced bioavailability. On the other hand, compound **14**, which also reduces the viral titer in 2-logs, follows the Lipinski’s rules and has a reasonably good predicted safety profile of administration in rats. However, further validation through toxicological reporting will be needed as well as studies of lead optimization, product formulation and efficacy.

## 5. Conclusions

In conclusion, our results underline the potential of the ferruginol derivatives and analogues as antivirals, and particularly in this case of in the treatment of coronavirus infections. They are accessed in short synthetic sequences in good overall yield from either the easily available methyl dehydroabietate, via commercial (−)-abietic acid, or commercial (+)-dehydroabietylamine. They do have druggable features encouraging additional studies of the mechanism as well other activities in related viruses, as they have demonstrated broad spectrum antiviral properties.

## Figures and Tables

**Table 1:** Molecular properties (drug-likeness) by Molinspiration online software for compounds **1**, **3** and **12**–**16** ^a,b^.

Compound	miLog *P*	MW	n-HBA	n-HBD	TPSA	Lipinski’s Violation
**1**	6.41	286.46	1	1	20.23	1
**3**	7.08	431.58	4	1	59.30	1
**12**	5.24	302.46	2	2	40.46	1
**13**	4.33	316.44	3	2	57.53	0
**14**	4.67	301.47	2	3	46.25	0
**15**	6.88	449.57	4	1	59.30	1
**16**	6.25	449.57	4	1	59.30	1
**Rule of five**	not >5	<500	not >10	not >5		1 violation allowed

^a^ Values were calculated using Molinspiration Cheminformatics Online 2023 software (Molinspiration, accessed on 27 January 2023, Slovensky Grob, Slovak Republic, 2023, http://www.molinspiration.com). ^b^ P = partition coefficient; MW = molecular weight; n-HBA = number of hydrogen bond accepting groups; n-HBD = number of hydrogen bond donating groups; TPSA = total polar surface area.

**Table 2:** Acute rat toxicity prediction by GUSAR (on the Basis of PASS Prediction).

Acute Rat Toxicity Parameters	Compounds						
	1	3	12	13	14	15	16
	Rat LD_50_ Values						
**Rat IP ^a^ LD_50_ (mg/kg)**	0.00	1820.00	847.50	807.50	607.20	567.60	657.60
**Rat IV ^b^ LD_50_ (mg/kg)**	0.00	30.90	33.52	34.50	17.84	24.57	32.71
**Rat Oral ^c^ LD_50_ (mg/kg)**	0.00	1015.00	2175.00	2639.00	2167.00	1385.00	1429.00
**Rat SC ^d^ LD_50_ (mg/kg)**	0.00	400.80	602.90	295.00	882.00	227.30	420.30
**Acute rodent toxicity classification of compounds by OECD project**							
**Rat IP LD_50_ Classification**	Class 1	Non-toxic	Class 5	Class 5	Class 5	Class 5	Class 5
**Rat IV LD_50_ Classification**	Class 1	Class 1	Class 3	Class 3	Class 3	Class 3	Class 3
**Rat Oral LD_50_ Classification**	Class 1	Class 4	Class 5	Class 5	Class 5	Class 4	Class 4
**Rat SC LD_50_ Classification**	Class 1	Class 4	Class 4	Class 4	Class 4	Class 4	Class 4

^a^ IP—Intraperitoneal route of administration ^b^ IV—Intravenous route of administration ^c^ Oral—Oral route of administration ^d^ SC—Subcutaneous route of administration.

## Data Availability

The data is available in the manuscript and the Supporting Information of this article. Raw data are available upon request.

## Supplementary Materials

The following supporting information can be downloaded at: https://www.mdpi.com/article/10.3390/v15061342/s1, Figures S1–S8: ^1^H, ^13^C, DEPT and ^19^F NMR spectra for compounds **15** and **16**.
