# Supplementary material for: Anticoronavirus Evaluation of Antimicrobial Diterpenoids: Application of New Ferruginol Analogues
Source: Viruses. 2023 Jun 9;15(6):1342. doi: 10.3390/v15061342 (PMC10301393; doi:10.3390/v15061342)
Supplement: Supplementary file 1 [file viruses-15-01342-s001.zip › viruses-2431923-supplementary.pdf]

Supplementary Materials

Anticoronavirus Evaluation of Antimicrobial Diterpenoids: Application of New Ferruginol Analogues

Mihayl Varbanov [1,2], Stéphanie Philippot [1] and Miguel A. González-Cardenete[3,*]

[1] Université de Lorraine, CNRS, L2CM, F-54000 Nancy, France.

[2] Laboratoire de Virologie, CHRU de Nancy Brabois, 54500 Vandoeuvre-lès-Nancy, France

[3] Instituto de Tecnologia Química (UPV-CSIC), Universitat Politècnica de Valencia-Consejo Superior de Investigaciones Científicas, Avenida de los Naranjos s/n, 46022 Valencia, Spain

* Correspondence: migoncar@itq.upv.es

Contents

Copies of ^{1}H NMR , ^{13}C NMR and ^{19}F spectra for new compounds **15** and **16**:

-^{1}H NMR spectrum of **15**……………...………..………………………………………S1

-^{13}C NMR spectrum of **15**………..…..……………………………………………S2

-DEPT135 spectrum of **15**…………………………………………………………S3

-^{19}F spectrum of **15**……………………………………………………………...S4

-^{1}H NMR spectrum of **16**……………...………..………………………………………S5

-^{13}C NMR spectrum of **16**………..…..……………………………………………S6

-DEPT135 spectrum of **16**…………………………………………………………S7

-^{19}F spectrum of **16**……………………………………………………………...S8

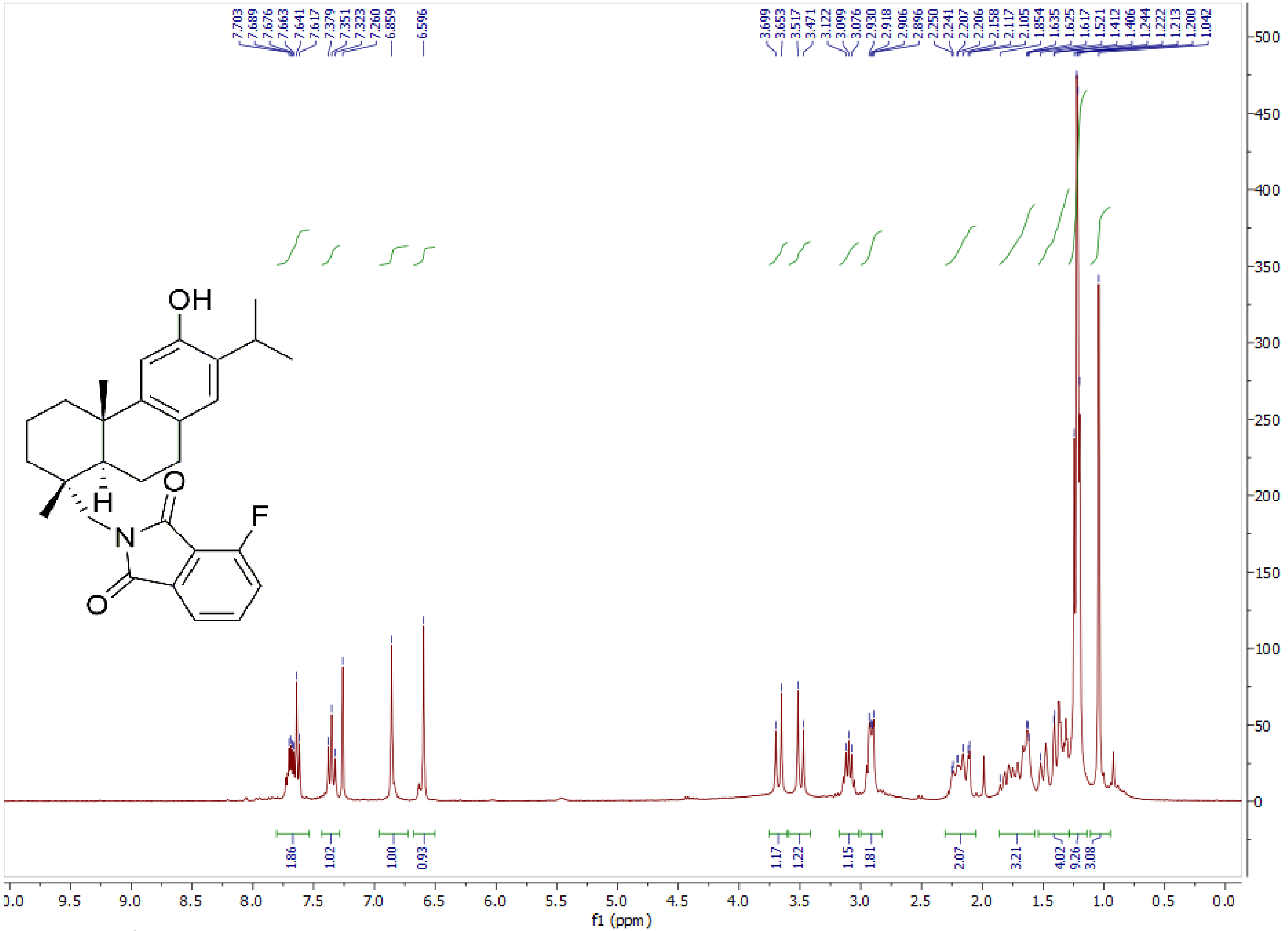

Figure S1. ^{1}H NMR spectrum of **15**.

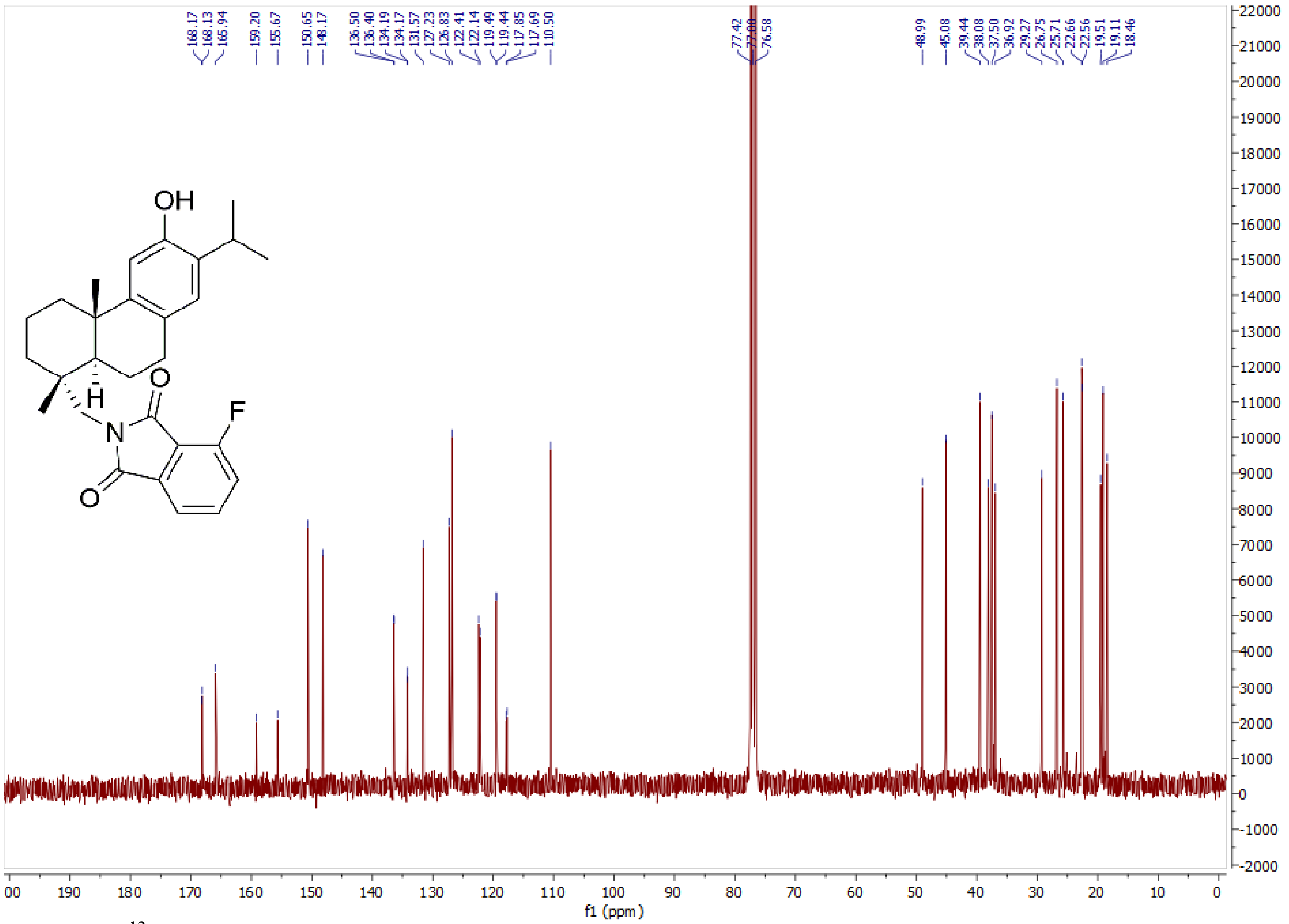

Figure S2. ^{13}C NMR spectrum of **15**.

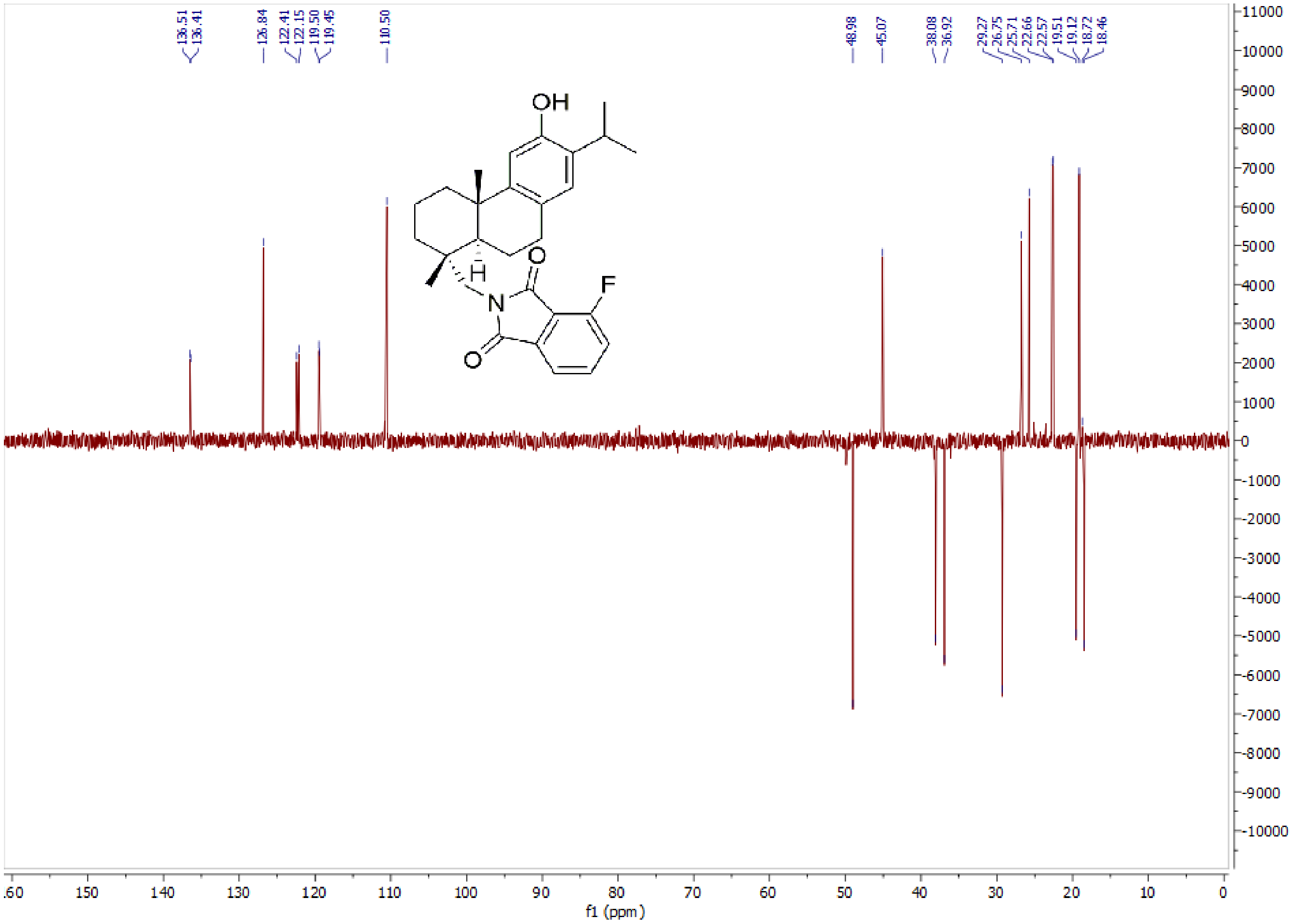

Figure S3. DEPT135 spectrum of **15**.

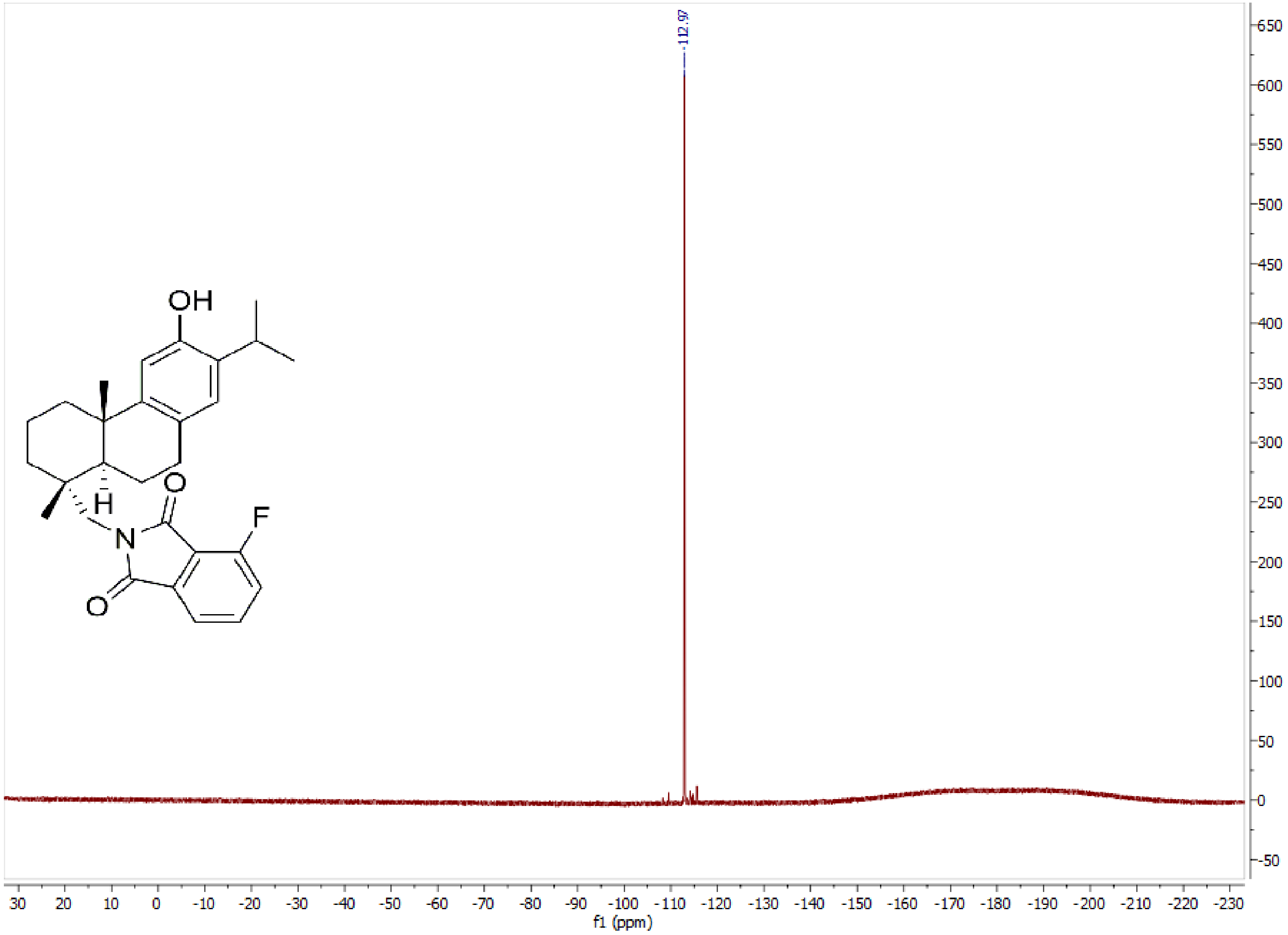

Figure S4. ^{19}F spectrum of **15**.

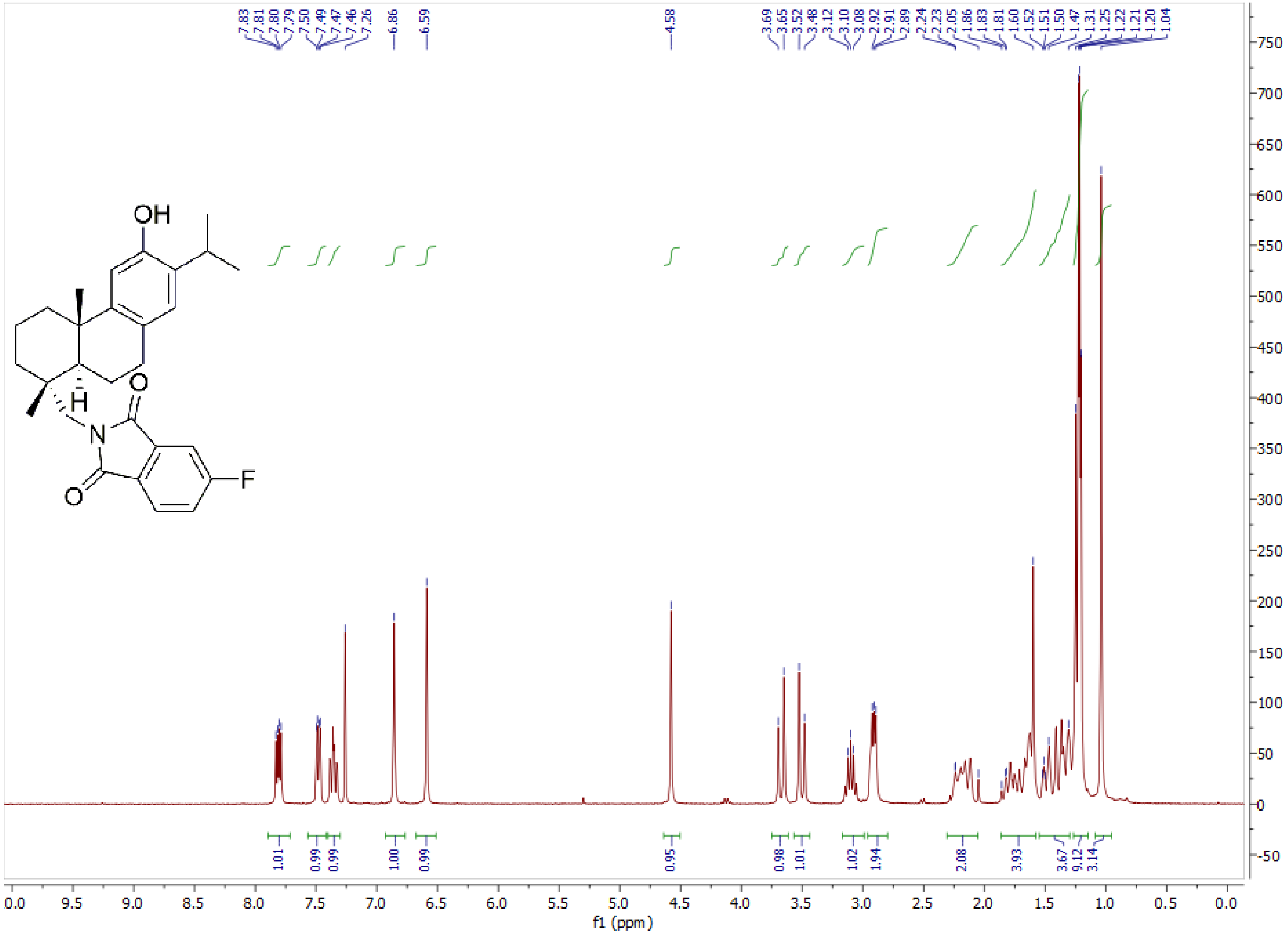

Figure S5. ^{1}H NMR spectrum of **16**.

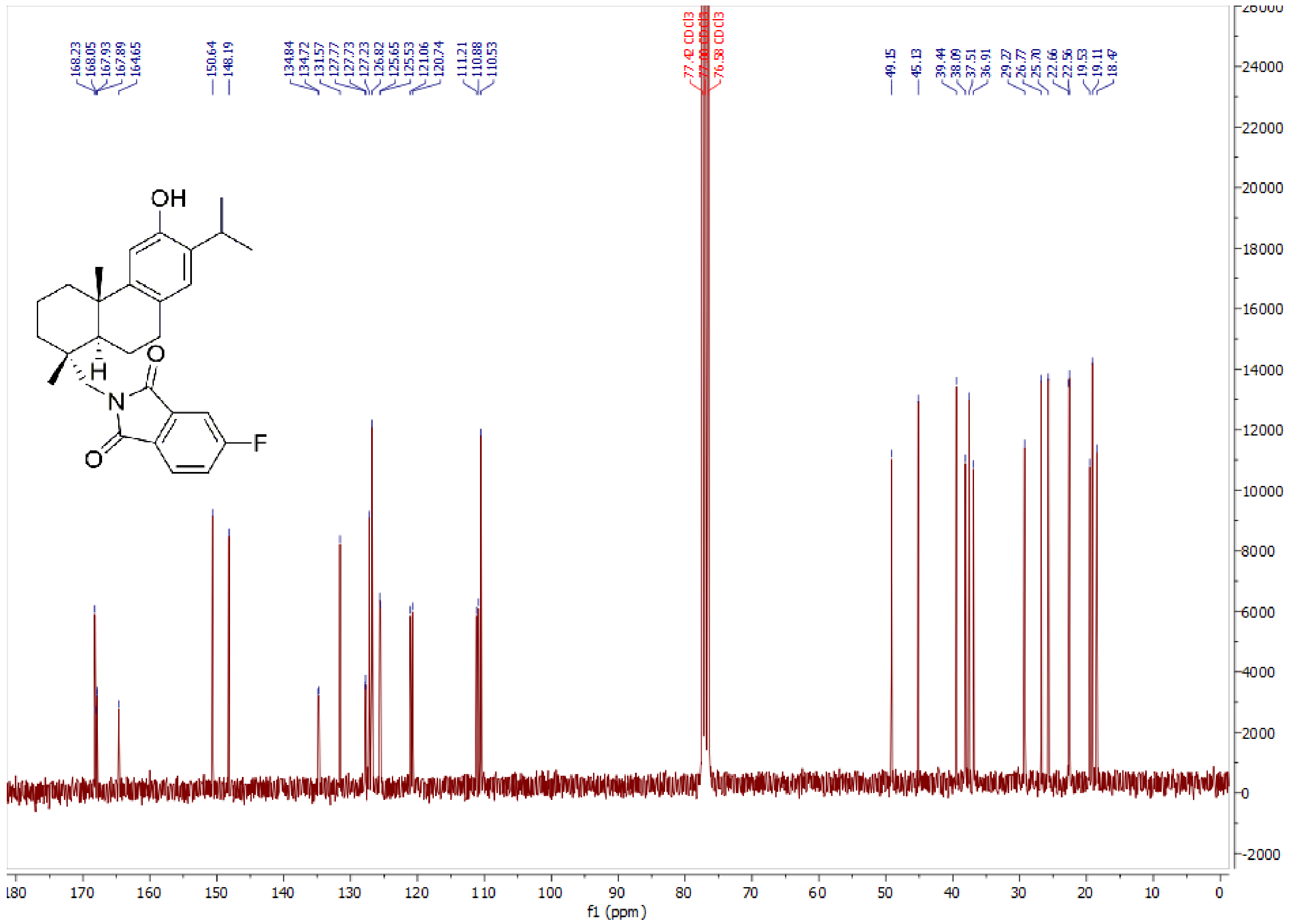

Figure S6. ^{13}C NMR spectrum of **16**.

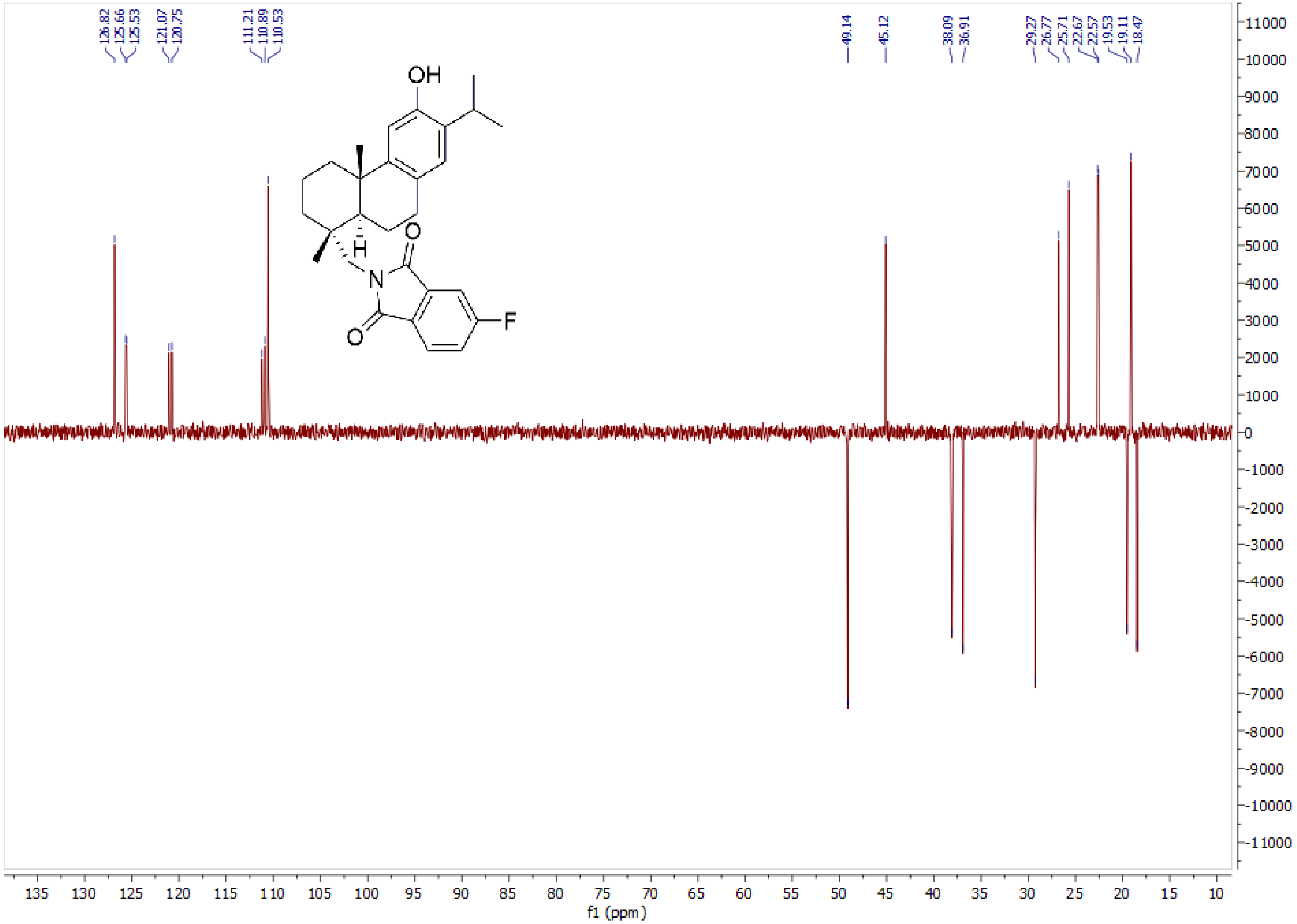

Figure S7. DEPT135 spectrum of **16**.

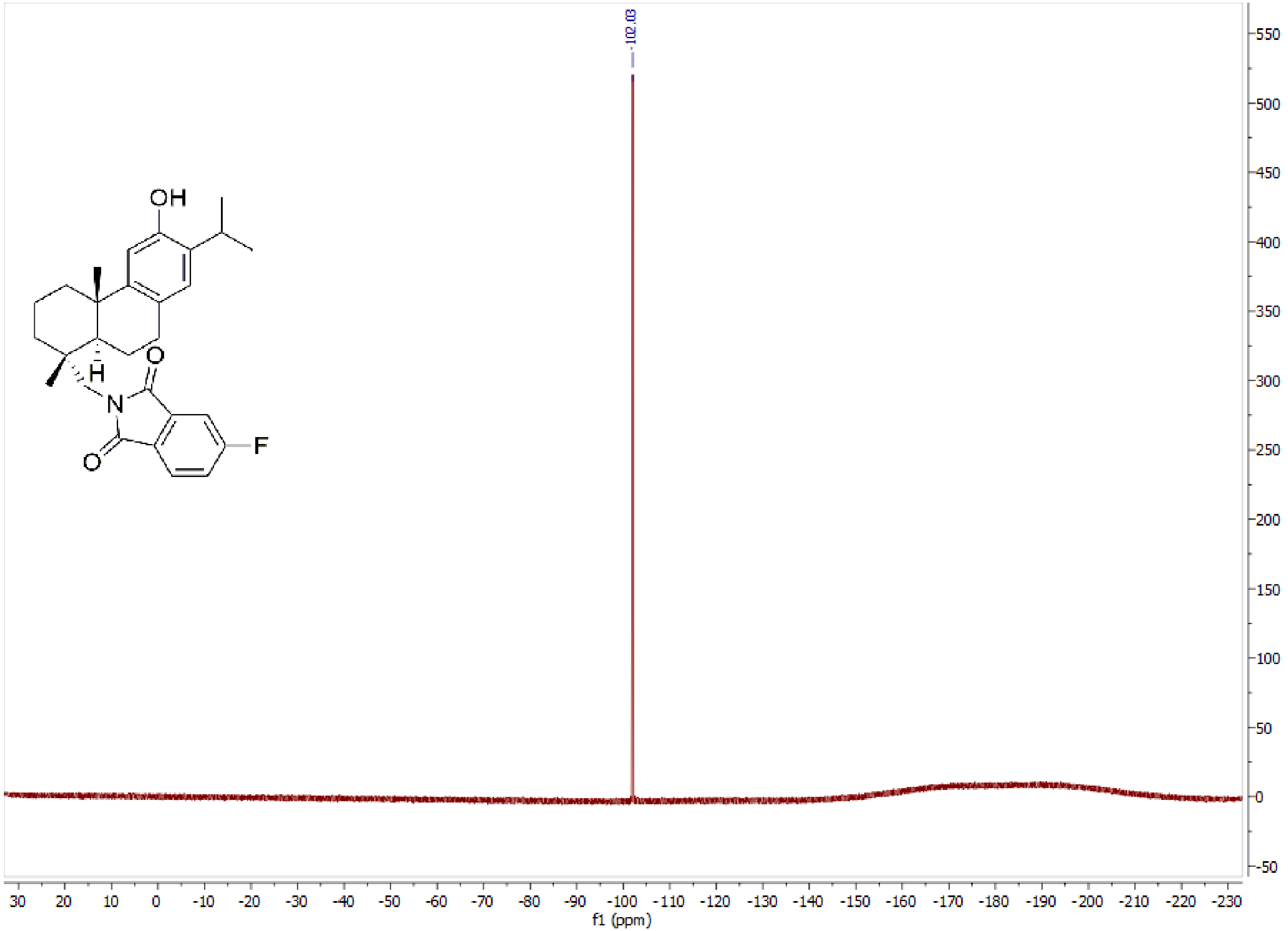

Figure S8. ^{19}F spectrum of **16**.
